# Supplementary material for: The Atlas of Inflammation Resolution (AIR)
Source: Mol Aspects Med. 2020 Aug;74:100894. doi: 10.1016/j.mam.2020.100894 (PMC7733955; doi:10.1016/j.mam.2020.100894)
Supplement: Multimedia component 1 [file mmc1.docx]

**Supplementary Table 1.** List of key submaps available in the AIR.

| **Phase** | **Submap title** |
| --- | --- |
| Initiation | Pathogen associated molecular pattern (PAMPs) signaling |
|  | Vasodilation vasoconstriction and permeability |
|  | Leukocyte adhesion and transmigration |
|  | Neutrophil chemotaxis |
|  | Natural killer cell chemotaxis |
|  | Biosynthesis of prostaglandins, thromboxanes and leukotrienes from arachidonic acid |
|  | Biosynthesis of prostaglandins from EPA |
| Transition | Neutrophil apoptosis |
|  | Macrophage phagocytosis |
|  | Monocyte transmigration |
|  | Efferocytosis |
| Resolution | Macrophage M1 to M2 class switch |
|  | Biosynthesis of lipoxins from arachidonic acid |
|  | Biosynthesis of resolvins, maresins, protectins and their conjugates from DHA |
|  | Biosynthesis of E-class resolvins and lipoxins from EPA |
|  | Resolvins mediated signaling cascade |
|  | Protectins mediated signaling cascade |
|  | Maresins mediated signaling cascade |
|  | Lipoxins mediated signaling cascade |
|  | STOP signaling |
| Homeostasis and return to tissue function | Wound healing |
